# Dynamically Tunable Phase Shifter with Commercial Graphene Nanoplatelets

**DOI:** 10.3390/mi11060600

**Published:** 2020-06-20

**Authors:** Muhammad Yasir, Patrizia Savi

**Affiliations:** Department of Electronics and Telecommunications, Politecnico di Torino, 10129 Torino, Italy; muhammad.yasir@polito.it

**Keywords:** commercial graphene nanoplatelets, tunable microwave devices, phase shifter, voltage controlled microwave components

## Abstract

In microwave frequency band the conductivity of graphene can be varied to design a number of tunable components. A tunable phase shifter based on commercial graphene nanoplatelets is introduced. The proposed configuration consists of a microstrip line with two stubs connected with a taper. On each side of the stubs there is a gap, short circuited through a via, where the commercial graphene nanoplatelets are drop casted. By applying a DC bias voltage that alters the graphene resistance the phase of the transmitted signal through the microstrip line can be varied. In order to maximize the phase shift of the transmitted signal and minimize the insertion loss, the length of the taper and the stubs are optimized by the help of circuit model and full-wave simulations. A prototype working at 4GHz is fabricated and measured. A phase variation of 33 degrees is acquired with an amplitude variation of less than 0.4 dB.

## 1. Introduction

Among the various carbon based materials, graphene is the most notable [1,2,3]. Due to its interesting properties graphene has caught significant attention [4]. Not only has graphene been studied in its original form but also for functionalization and patterning [5,6]. One of the most popular deposition methods of carbon based materials is their deposition as films [7,8]. The advantage of using graphene is that a lot of research work has been performed on its fabrication techniques that has facilitated its production and reduced its cost over the years. This has incentivized its widespread use in a number of different applications. 

Graphene has remarkable electrical, mechanical and thermal properties. Due to the remarkable properties of graphene, it has found inwards into several applications including electrochemical sensors [8,9], biosensors [10,11], gas sensors [12,13,14], humidity and temperature monitoring [15,16,17], absorbing materials [18], passive [19,20,21] and active devices [22,23]. The electrical properties of graphene vary with frequency. Due to the occurrence of plasmonic effect in graphene at the terahertz frequency range, it has been deeply analyzed [24,25]. In the microwave frequency band, graphene has emerged only recently in components as tunable phase shifter [26], attenuators [27,28,29] and antennas [30,31]. It has been noted that graphene varies its electron mobility with the application of a DC voltage. The variation of electron mobility results in taking the Fermi energy level from conduction to valence band. This makes graphene from a highly insulative material to a considerably conductive material. This variation of conductivity with the application of a DC voltage is valid through a wide frequency band covering the entire microwave frequency spectrum. 

Communication systems involve a number of components working at different frequencies. For efficient working, there needs to be an interconnection between components that form an entire communication system. This interconnection can be facilitated if the components are able to tune their working frequency. Therefore, the tuning of microwave components that form a communication system is vital to efficient functionality. Graphene being tunable with the application of a DC bias, is a good contender for being deployed in microwave communication systems. The acquisition of monolayer graphene is technologically demanding and not very cost effective. Multilayer graphene on the other hand bears tunable conductive behavior similar to monolayer graphene albeit with reduced cost and very low technological complexity. Until recently, multilayer graphene has been grown in laboratory. The availability of commercial graphene nanoplatelets on a large scale takes the ease of fabrication and commercialization of tunable components based on graphene one step further. Recently, a tunable attenuator [29] and antenna [30] have been realized exploiting the tunable conductivity of commercial graphene nanoplatelets. 

In this paper, a tunable phase-shifter based on commercial graphene nanoplatelets is designed. The proposed configuration consists of a microstrip line with two stubs connected with a taper. On each side of the stubs there is a gap, short circuited through a via, where the commercial graphene nanoplatelets are drop casted. By applying a DC bias voltage that alters the graphene resistance the phase of the transmitted signal through the microstrip line can be varied. The lengths of the tapered line and open line sections are optimized by the help of a circuit model. The phase shifter is further optimized with a full-wave simulator. A prototype of the tunable phase shifter is fabricated and measured. A variable phase shift of 33 degree is obtained with a degradation of the insertion loss of less than 0.4 dB. 

## 2. Materials and Methods 

### 2.1. Graphene Characterization

The type of graphene used in this work is graphene nanoplatelets based on multiple graphene layers. The graphene nanoplatelets are produced by Nanoinnova. Raman and FESEM (Field Emission Scanning Probe Microscope) are used for morphological characterization of the graphene nanoplatelets. 

**Figure 1:**
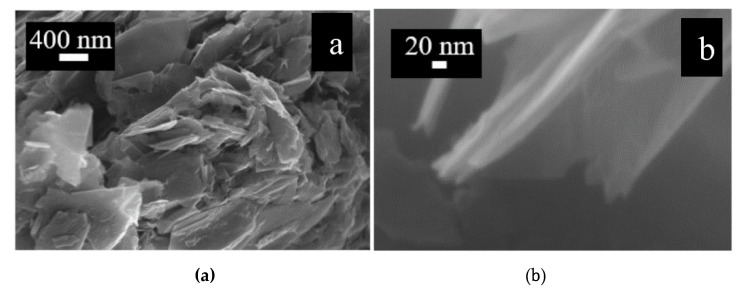
FESEM images of the commercial graphene nanoplatelets. (**a**) multiple graphene flakes (**b**) individual flake.

For FESEM analysis of the commercial graphene nanoplatelets, a ZEISS SUPRA ^TM^ 40 microscope was used. The FESEM images are shown in Figure 1. In Figure 1a, a zoomed out image of the graphene nanoplatelets can be seen. A zoomed in image of a single graphene nanoplatelet is shown in Figure 1b. The transparency of flake at such a scale shows that the thickness of the individual flake is a few nanometers and hence is composed of only a few graphene layers. 

**Figure 2:**
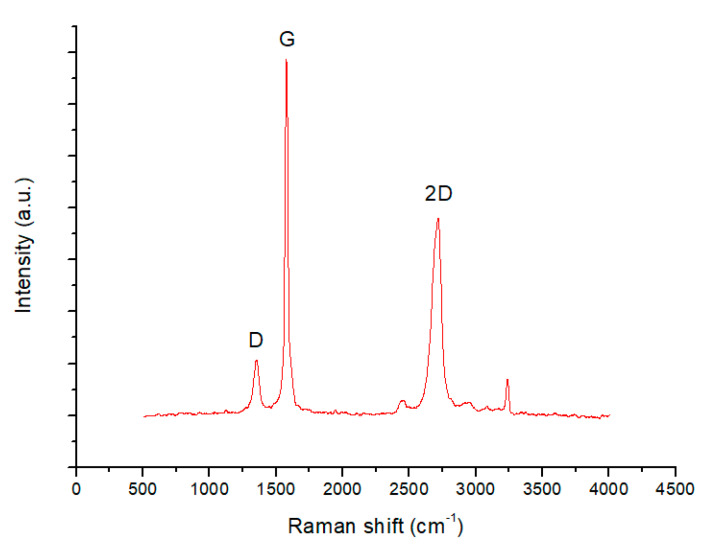
Raman spectroscopy of the commercial graphene nanoplatelets.

Raman spectroscopy performed on the graphene nanoplatelets is shown in Figure 2. There are two different spectral ranges for characterizing the Raman spectrum of graphene. The first one in the range of 1000–1700 cm^−1^ contains the D (defects) and G (graphitization grade). The second spectral range from 2200–3500 cm^−1^ is the second order Raman spectral range containing overtones. The ratios of the peaks are I_D_/I_G_ = 0.15, I_D_/I_2D_ = 0.27 and I_G_/I_2D_ = 1.81. According to guidelines described in [32], the Raman spectroscopy shows a similar behavior to that of few layer graphene. A detailed analysis of the relation between the intensities and shape of the peaks G and 2D to the number of graphene layers can be found in [4]. In the case of monolayer graphene I_2D_/I_G_ ≥ 1 and there is no broadening in the feature of the 2D band. For the commercial graphene used here, the shape of the 2D is slightly broadened and I_2D_/I_G_ = 0.55. This shows that the graphene nanoplatelets used comprise of a number of graphene layers.

### 2.2. Circuit Model Optimization

**Figure 3:**
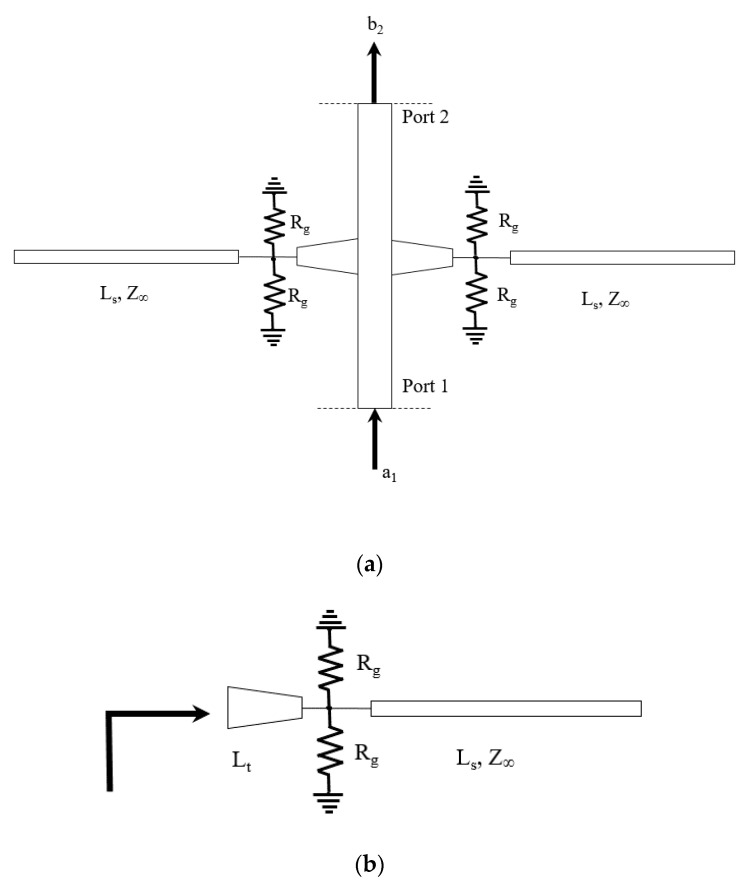
(**a**) Two-port phase shifter circuital representation (**b**) Circuital representation of the stub.

The phase shifter is composed of a microstrip line connected to two stubs (see Figure 3a). For a two-port device the transmission properties are defined by the scattering parameter S_21_ = b_2_/a_1,_ where b_2_ is the signal transmitted at Port 2 with a_1_ incident at Port 1. S_21_ is a complex number and can be represented as either real and imaginary part, or amplitude and phase.

The stubs are composed of a linear tapered line and an open line section connected to each other through grounded resistors, representing graphene as shown in Figure 3. The tapered line has length, L_t_ and thickness corresponding to a characteristic impedance of 50 Ω at one end. The other end of the tapered line, which is connected to the stub, has a thickness corresponding to 100 Ω. The tapered line reduces reflection from the open line section that has a characteristic impedance of 100 Ω and length L_s_. Graphene is modelled as a lumped resistor with resistance R_g_. 

The current passing into the stub is controlled through the graphene’s resistance. A higher resistance of graphene means that the impact of the stub is maximized and so is the total reactance as seen at the input. A lower graphene’s resistance means a maximum current passing through graphene into the ground minimizing the impact of the stub. The input impedance of the stub structure is given by Z_in_ = R_in_ + jX_in_. This is composed of a real part, R_in,_ and an imaginary part, X_in_. When the graphene’s resistance varies both the values of R_in_ and X_in_ varies. It is desirable to maximize the variation of X_in_ and minimize the variation of R_in_ when the graphene resistance, R_g_ is varied. The lengths of the tapered line, L_t_ and the open line section, L_s_ is therefore optimized for a maximum X_in_ and minimum R_in_ variation when graphene’s resistance R_g_ is varied. 

Simulations are performed based on the circuit model shown in Figure 3 at a frequency of 4 GHz. The length L_t_ is varied from 0.04 λ_0_ to 0.08 λ_0_ and the length L_s_ is varied from 0.05 λ_0_ to 0.35 λ_0_ where λ_0_ = c/f, c is the speed of light and f is the frequency used in the design (4 GHz).

For each set of values of L_t_ and L_s_, the input impedance is simulated for values of R_g_ ranging from 70 Ω to 700 Ω. The values ΔR_in_ = R_in_ [R_g_ = 700 Ω]-R_in_ [R_g_ = 70 Ω] and ΔX_in_ = X_in_ [R_g_ = 700 Ω]-X_in_ [R_g_ = 70 Ω] are found as shown in Table 1. It can be observed that by increasing the length, L_s_ the value of ΔX_in_ is increased while increasing the value of L_t_, the value of ΔR_in_ is reduced up to length L_s_ = 0.3 λ_0_. Increasing the length, L_s_ further reduces the value of both ΔR_in_ and ΔX_in_. For the case of L_s_ = 0.15λ_0_, in which there is no impact of the variation of R_g_ on Z_in_. The best case from this analysis is thus: L_s_ = 0.3 λ_0_ and L_t_ = 0.08 λ_0_.

### 2.3. Full-Wave Design 

**Figure 4:**
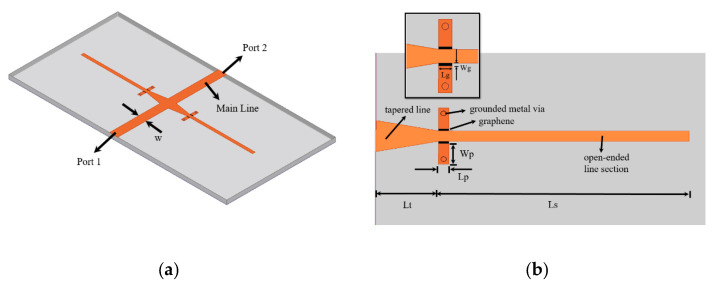
Geometrical representation of the phase shifter with dimensions: (**a**) phase shifter; (**b**) individual stub.

The operating principle of the phase shifter is to have a variable reactance on a transmission line caused by the optimized stubs of the Section 2.2. In order to achieve considerable phase variation, two stubs are connected to a 50 Ω transmission line forming a two-port structure. A geometrical representation of the phase shifter is shown in Figure 4. The phase shifter is designed on a Rogers 3035 dielectric substrate of thickness t = 1.52 mm. The dielectric permittivity of the substrate is ε_r_ = 3.5 and loss tangent is tanδ = 0.0015. The thickness of copper is 35 μm. The width of the main line is w = 3.2 mm, corresponding to a characteristic impedance of 50 Ω. The stub is shown in detail in Figure 4b. The stub is composed of a tapered line section of length, L_t_ and an open ended line section of length, L_s_. In order to realize the grounds, two grounded metallic vias are symmetrically placed on each side of the line section in the middle of a metallic pad. The metallic pad has length, L_p_ = 1 mm and width, w_p_ = 2 mm. In between the metallic pad and the line section, graphene is deposited. The length of graphene deposition is equal to the length of the metallic pad, L_p_ = 1 mm. The graphene deposition has width, W_g_ = 0.2 mm and length, Lg = 1 mm. The aspect ratio is kept low to ensure lower resistance since commercial graphene nanoplatelets possess higher sheet resistance value. 

**Figure 5:**
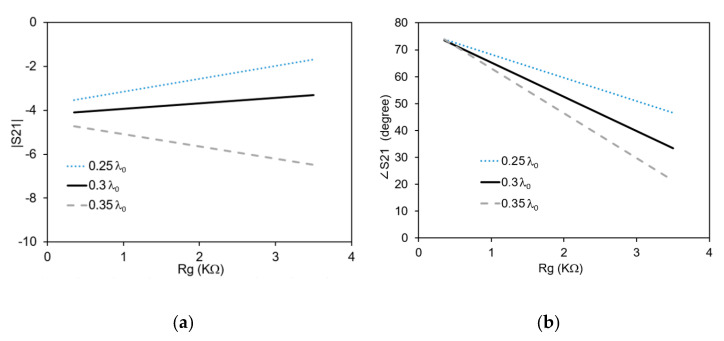
S_21_ versus R_g_ for different L_s_: (**a**) amplitude variation; (**b**) phase variation.

The phase shifter is simulated with a full-wave simulator Ansys HFSS. In order to further optimize the structure, the phase shifter has been simulated at a frequency of 4 GHz with three different lengths of the open-ended line section, L_s_ (0.25 λ_0_, 0.3 λ_0_ and 0.35 λ_0_) for graphene resistance values ranging between 350 Ω/sq. and 3500 Ω/sq. (the graphene sheet resistance is measured in Ohm/square). The amplitude and phase variation of the phase shifter versus graphene sheet resistance are shown in Figure 5. The amplitude variation of the transmission (see Figure 5a) as seen from the slope of the curves, decreases from L_s_ = 0.25 λ_0_ to L_s_ = 0.3 λ_0_. Increasing the value of L_s_ further to 0.35 λ_0_ results in increased variation of |S_21_|. The phase variation is shown in Figure 5b. It can be seen that the variation of ∠S_21_ increases with increasing L_s_. The maximum phase variation is attained in the case of L_s_ = 0.35 λ_0_. The optimum length is L_s_ = 0.3 λ_0_ because it provides minimum amplitude variation with reasonable phase variation.

### 2.4. Prototype Realization

**Figure 6:**
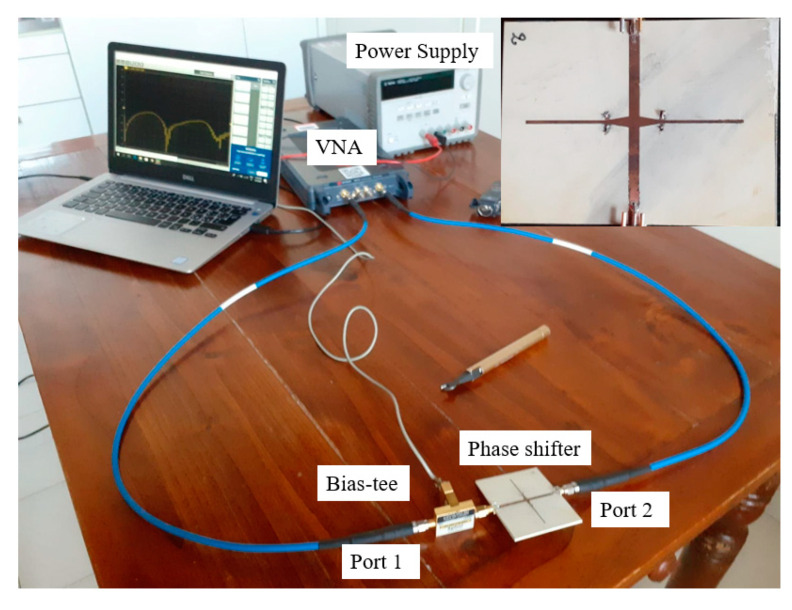
Measurement setup of the commercial graphene based tunable phase shifter. In the inset a photograph of the prototype is shown.

The structure of the phase shifter with optimized dimensions resulting from Section 2.2 and Section 2.3 is realized by using a standard etching process. Lithographic film is used to pattern the structure of the phase shifter on a dielectric substrate with both sides covered with copper. The substrate with the pattern is then immersed in acid to etch away excess copper. The metal vias are realized by drilling holes and soldering metal wires to the top and bottom. Commercial graphene nanoplatelets mixed in isopropyl alcohol are then drop casted on the designated spots of the phase shifter. The excess alcohol evaporates leaving behind the commercial graphene nanoplatelets. The fabricated prototype is as shown in Figure 6. 

## 3. Results

### 3.1. Full-Wave Simulations

**Figure 7:**
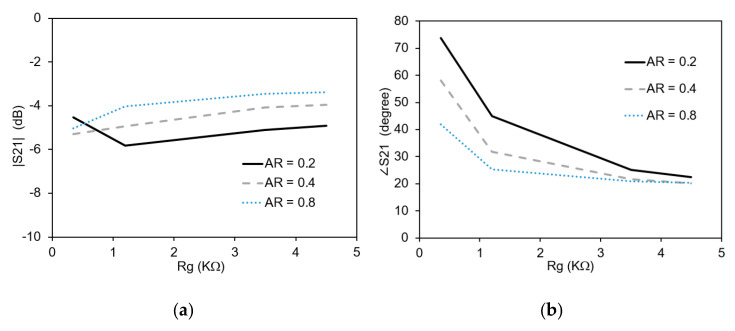
Impact of the aspect ratio on the transmission: (**a**) Amplitude variation; (**b**) Phase variation.

The aspect ratio of the gap with graphene can be defined as: AR = Wg/Lg (see Figure 4b inset). This is an important parameter in the determination of the resistance R = Rg AR. The value of the resistance, R can also be calculated from the ratio of the applied bias voltage and the current drawn by graphene [19]. In order to evaluate the impact of the aspect ratio on the transmission properties of the phase shifter, full-wave simulations are performed with different values of the aspect ratio ranging from 0.2 to 0.8 at a frequency of 4 GHz. The resultant amplitude and phase variation versus graphene sheet resistance, Rg, is shown in Figure 7. The reduction of AR causes a reduction in the variation of |S_21_|. For an AR of 0.2, the maximum and minimum |S_21_| is −4.5 dB and −5.8 dB respectively. For the maximum AR value of 0.8, the maximum and minimum |S_21_| is −3.3 dB and −5.0 dB respectively. The value of the phase of the transmission coefficient increases with a reduction in the aspect ratio. The maximum value of ∠S_21_ for an AR of 0.2 is 73° while the minimum value is 22°. This shows that a reduction of the AR reduces the variation of the amplitude of the transmission coefficient and increases the variation of the phase of the transmission coefficient, a highly desirable trait of tunable phase shifters. 

**Figure 8:**
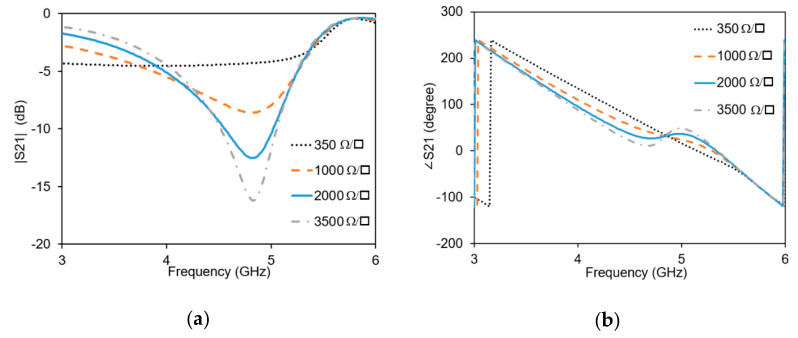
Simulated transmission with different graphene resistance: (**a**) amplitude shift; (**b**) Phase shift.

The optimized phase shifter is simulated with full-wave simulator in the frequency range of 3–6 GHz. Graphene nanoplatelets are modelled as infinitely thin resistive sheets with assigned resistance values ranging from 350 Ω/sq. to 3500 Ω/sq. The resulting simulated values of the transmission coefficient are shown in Figure 7. The amplitude of S_21_ (Figure 8a) reduces from 4 GHz to 5 GHz for higher resistance values. At the frequency of 4.3 GHz, the amplitude variation is minimum. The phase variation (Figure 8b) increases slightly from 3 GHz to 5 GHz. 

### 3.2. Measurements

**Figure 9:**
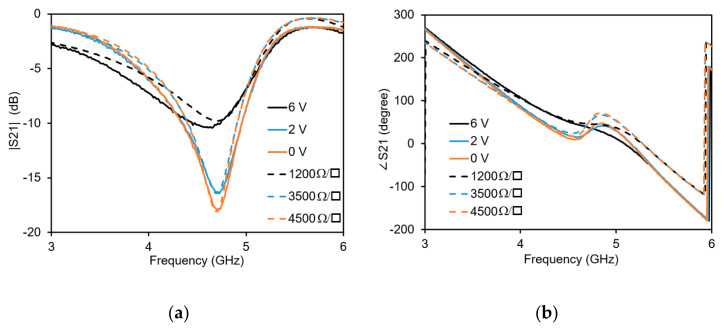
Transmission coefficient with measured (solid lines) and simulated values (dashed lines): (**a**) Amplitude; (**b**) Phase.

The measured results of the transmission are shown in Figure 9. Measurements of the prototype are carried out by the help of a vector network analyzer. A commercial broadband bias-tee is used to bias the commercial graphene nanoplatelets. The bias is applied between the ground plane and the main line. By varying the bias voltage applied to the commercial graphene nanoplatelets, their resistance is varied. This causes a variation of the phase of the signal transmitted between the two ports. At a minimum applied bias voltage of 0 V, the graphene resistance is 4500 Ω/sq. Increasing the bias voltage to 6 V results in reducing the graphene resistance to 1200 Ω/sq. The corresponding sheet resistance values as derived from the aspect ratio are 4500 Ω/sq., 3500 Ω/sq. and 1200 Ω/sq. respectively.

In order to compare measured and simulated values, simulations are performed with graphene sheet resistance values corresponding to the measured graphene resistance values. The simulated amplitude (dashed lines) of the S_21_ is shown in Figure 9a and the simulated phase (dashed lines) of S_21_ is shown in Figure 9b. It can be seen that the simulated and measured S_21_ are in good agreement with each other. For similar measured and simulated values of graphene, similar amount of phase and amplitude variation is observed. The maximum Figure of Merit (FoM = phase shift variation (degree)/ insertion loss variation (dB)) is observed at a frequency of 4.3 GHz. The measured phase shift at the frequency of 4.3 GHz is almost 33 degrees with a measured amplitude variation less than 0.4 dB. Hence the maximum figure of merit is 82.5 degree/dB.

**Figure 10:**
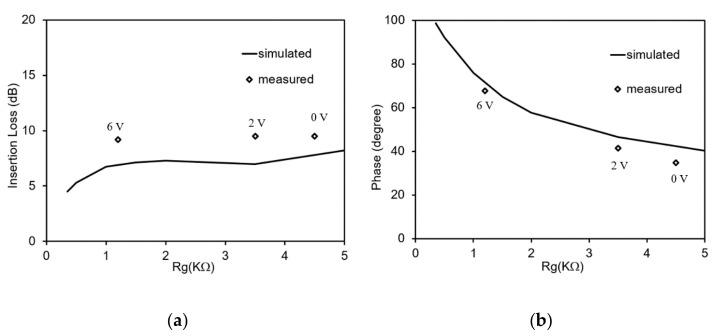
Measured and simulated results at 4.3 GHz: (**a**) Insertion loss versus Rg; (**b**) Phase versus Rg.

At this frequency the insertion loss and phase variation are simulated for different Rg values as shown in Figure 10. The measured insertion loss and phase are indicated as diamonds and marked by the voltage applied to the graphene deposition. The phase of the simulated and measured transmission coefficient are in good agreement with each other. Due to losses that are not totally taken into account in the simulated results, there is a slight difference between the simulated and measured insertion loss.

## 4. Discussion

The phase shifter presented is a dynamically tunable phase shifter that varies its phase upon an application of a voltage bias. The phase shifter deploys commercial graphene nanoplatelets and is a step towards mass-production of tunable microwave components based on graphene. The phase shifter provides almost 33 degrees of phase shift with negligible variation of the insertion loss. A comparison of the phase shifter with other similar phase shifters based on novel materials is shown in Table 2. In comparison to other phase shifters, the phase shift of the commercial graphene phase shifter is slightly lower but the variation of the insertion loss is negligible. This results in a higher figure of merit as compared to similar phase shifters. The phase shifter works really well at the designed frequency with minimal variation of the insertion loss and is thus suitable for deployment in steerable antennas. For an array comprising of 2 patch antennas spaced half a wavelength, this phase shift can produce a beam steering of almost 10 degrees. 

As shown in Section 3.2 the simulated results compared to the measured results show a slightly smaller phase and amplitude variation. This is due to a higher graphene sheet resistance value. The fabrication process of the phase shifter is of preliminary nature and needs to be improved for a more gradual variation of the phase with more voltage points. In addition, the commercial graphene nanoplatelets can be sonicated in order to reduce the number of graphene sheets per nanoplatelet and an increased variation of the graphene resistance and to obtain a smaller value. This would result in the possibility of depositing graphene in a gap with a higher aspect ratio and further ease the fabrication process. If a higher phase shift is desired, the number of the stubs can be further increased to 3 or 4. This can result in further increasing the beam steering value. 

The effects of a negative bias applied to the graphene nanoplatelets are predicted to change the carrier charge, the Fermi level and the electron mobility [4] resulting in an increase in the conductivity. This is a behavior similar to the one noted when applying a positive bias voltage.

## 5. Conclusions

A voltage controlled dynamically tunable phase shifter based on commercial graphene nanoplatelets is presented. The phase shifter is composed of a microstrip transmission line connected to a tapered line and an open stub. Graphene connected to grounded metallic vias are symmetrically placed at the interconnection between the tapered line and the stub. The electrical conductivity of graphene is tuned by a voltage bias, which results in the variation of the insertion loss and phase of the signal transmitting through the microstrip line. In order to maximize the phase shift and minimize the insertion loss, optimization of the lengths of the open stub, tapered lines and the dimensions of the graphene’s depositions are performed by the help of circuit models and full wave simulations. A prototype is fabricated and measured. The measured phase shift of the phase shifter is almost 33 degrees with a variation of the insertion loss of less than 0.5 dB at the frequency of 4.3 GHz. 

## Figures and Tables

**Table 1:** The variation of real and imaginary input impedance with graphene resistance variation with different values of L_s_ and L_t._ All ΔR_in_ and ΔX_in_ are in (Ω).

Ls	L_t_ = 3mm (0.04 λ_0_)		L_t_ = 4mm (0.053 λ_0_)		L_t_ = 5 mm (0.067 λ_0_)		L_t_ = 6 mm (0.08 λ_0_)	
	ΔR_in_	ΔX_in_	ΔR_in_	ΔX_in_	ΔR_in_	ΔX_in_	ΔR_in_	ΔX_in_
0.05 λ_0_	41.5	46.9	37.6	40	34.5	34.7	31.75	30.8
0.15 λ_0_	0.3	0.02	0.3	0.02	0.3	0.02	0.3	0.03
0.3 λ_0_	48	67	45	58	41	50	38	44
0.35 λ_0_	43	52.6	39	45	36.6	38	33.4	34.5

**Table 2:** Comparison of the commercial graphene based phase shifter with others in the literature.

Ref.	Δφ (°)	ΔIL (dB)	FOM (°/dB)
[26]	40	3	13.3
[33]	53.76	2	26.88
This work	33	0.4	82.5
